# A Whole Community Approach toward Child and Youth Resilience Promotion: A Review of Resilience Literature

**DOI:** 10.1007/s11469-013-9470-1

**Published:** 2014-01-15

**Authors:** Nazilla Khanlou, Ron Wray

**Affiliations:** 1 York University, HNES 3rd floor, 4700 Keele Street, Toronto, ON Canada M3J 1P3; 2 DGL Consulting, Toronto, ON Canada

**Keywords:** Resilience, Child, Youth, Mental Health Promotion, Social Determinants of Health, Health Equity, Literature Review

## Abstract

A literature review of child and youth resilience with a focus on: definitions and factors of resilience; relationships between resilience, mental health and social outcomes; evidence for resilience promoting interventions; and implications for reducing health inequities. To conduct the review, the first two following steps were conducted iteratively and informed the third step: 1) Review of published peer-review literature since 2000; and 2) Review of grey literature; and 3) Quasi-realist synthesis of evidence. Evidence from three perspectives were examined: i) whether interventions can improve ‘resilience’ for vulnerable children and youth; ii) whether there is a differential effect among different populations; and, iii) whether there is evidence that resilience interventions ‘close the gap’ on health and social outcome measures. Definitions of resilience vary as do perspectives on it. We argue for a *hybrid approach* that recognizes the value of combining multiple theoretical perspectives, epistemologies (positivistic and constructivist/interpretive/critical) in studying resilience. Resilience is: a) a process (rather than a single event), b) a continuum (rather than a binary outcome), and c) likely a global concept with specific dimensions. Individual, family and social environmental factors influence resilience. A social determinants perspective on resilience and mental health is emphasized. Programs and interventions to promoting resilience should be complimentary to public health measures addressing the social determinants of health. A whole community approach to resilience is suggested as a step toward closing the public health policy gap. Local initiatives that stimulate a local transformation process are needed. Recognition of each child’s or youth’s intersections of gender, lifestage, family resources within the context of their identity markers fits with a localized approach to resilience promotion and, at the same time, requires recognition of the broader determinants of population health.

Resilience is a term attracting wide and diverse interest from practitioners, educators, researchers and policy makers alike. Conceptual and empirical work and policy making is taking note of what has been called the ‘ordinary magic’ of many children and adolescents overcoming daunting social circumstances or traumatic life events (Masten 2001). Such heightened awareness cuts across different social policy sectors including health care, mental health, education, child and youth services, justice and even public security. Of course, few of us in this day and age believe in ‘magic’ despite the compelling attraction of such imagery. Instead, decades of work on the possible factors underlying the ‘magic’ of resilience, and an expanding base of evaluation research on interventions, is providing a series of explanations for how resilience might be fostered and sustained. For those committed to public health and social policy these advances in knowledge are pushing resilience from the realm of mystery to a population health focus and action.

Resilience is seen as an important element to maintaining and promoting child and youth mental health, and as a life-long buffer to potential threats to wellbeing over time and transition. It is a strength based concept that builds on individual’s strengths rather than emphasizing deficits. Investing in the resilience of individuals and investing early in the life course is seen as a powerful health promoting step with lifelong benefits including potential improvements in school, employment, and pro-social outcomes–as well as a possible ‘equalizer’ in socio-economic differences. Yet when exploring resilience it is perhaps best to clearly state up front what it is not. Resilience is not just a personality trait or attribute of an individual (Luthar and Cicchetti 2000; Masten et al. 1999). Rather, resilience is most often viewed as a process that refers to exposure to adversity and “positive” adaptation (Fergus and Zimmerman 2005; Luthar et al. 2000). Between these two points are a host of protective factors such as family, school, community and society that appear to modify vulnerability to the effects of adversity. And there will always be a need to acknowledge some will require specialized and on-going help through-out their life through no fault of their own.

In this review paper we explore how resilience initiatives and interventions might help to improve mental and physical health, foster social wellbeing, and reduce inequitable health status for those traditionally excluded for social or economic reasons. Specifically, the following questions are addressed: 1) what is the definition of resilience?; 2) what are the different perspectives and approaches used in the study of resilience; 3) what are the factors that are associated with the process of resilience and mental health for children and youth?; 4) what is the relationship of resilience to social outcomes (e.g. mental health, academic performance, health behaviours); and, 5) what is the research evidence that interventions designed to promote resilience and mental health are effective, with special attention to reducing the differences or gaps between children and youth in disadvantaged/excluded social conditions? The article concludes with a discussion of the implications for advancing positive mental health and the reduction of health inequities, and suggests the necessity of adopting a ‘whole community approach’ in which families, schools and local services work together. The paper is based on a review the authors conducted for the Public Health Agency of Canada–PHAC (Khanlou and Wray 2010). It reflects the views of the authors and not those of PHAC.

## Review Methods

### Peer Reviewed Literature Search Methodology–Post 2000

To identify relevant literature for the review, online searches were be conducted on databases, including MEDLINE (the National Library of Medicine’s database), HEALTHSTAR, CINAHL (Cumulative Index to Nursing and Allied Health Literature), PsychoINFO, PUBMED, Sociological Abstracts, and Psychological Abstracts. Publications from year 2000 to current were considered. However, seminal and relevant articles from previous years were also included. Combinations of keyword searches with Boolean operators (and/or/*/?) were used (such as resilience, social emotional learning, adolescence/youth, children, mental health promotion, schools). The initial focus was on Canadian based research, however, relevant studies from other countries such as the USA, Australia, the United Kingdom, and other countries in Europe were also consulted. Documents produced by the World Health Organization were also reviewed.

### Grey Literature Search Methodology

The initial starting point was governmental and research institute sites including international jurisdictions. The search process was further enhanced through personal contacts to identify additional grey literature, particularly those of local initiatives not regularly accessible through electronic search methods. As well, a systematic method of “snowballing” was employed to build upon learning from the first grey literature examination.

### A Strategy for the Synthesis of Evidence–Quasi-Realist Approach

To generate useable and reliable findings and knowledge that can be translated into a clear set of directions and interventions, it is necessary to adopt a systematic approach to appraise, interpret and synthesize literature from diverse sources. This requires the use of clearly formulated questions with explicit rules on assessing the literature and translating findings.

At the first level, the review and analysis of the material adopted a hybrid approach (i.e., individual factors, ecological/ecosystem and constructivist perspectives) to extract the most useable and reliable information. Such a hybrid approach maximizes the exploratory power and learning potential of both quantitative and qualitative material–a step considered critical by us when analyzing and synthesizing what is, to a great degree, an emerging policy field.

At the second level, a quasi-realist approach was utilized. Conceived, developed and implemented by Pawson and colleagues (2004), the realist approach has been regularly used to generate reliable findings for articulating how complex interventions function in different contexts. This emergent approach is founded on the recognition that most social and health issues are complex rather than simple, and an over reliance on the question ‘what works’ will alternatively risk discounting important information such as intervention design and process and, more critically, fail to yield important information on what works “for whom, under what circumstances, in what respects and how”? (Pawson et al. 2004). This requires moving beyond typical study design assessment criteria such as statistical power, sampling design and so on, by focusing on descriptive detail about intervention process and context. The “quasi-“approach reflects the fact that many programs and interventions reflect different population orientations as well as multi-level components, and many studies do not fully explain what program components are having the positive effect or the strength of the effect. Moreover, given the relative newness of the field of resilience initiatives, many programs and interventions are not necessarily categorized by the term resilience or other consistent terminology.

## Review Findings

### Definitions of Resilience

Despite the increasing frequency in the use of the term ‘resilience’, there is a surprising lack of consensus and clarity in the meaning and implications of the word. Origins of the concept of resilience in mental health field and within the western and industrialized context are attributed to studies of youth in the 20th century who having experienced significant adversity were observed to have good outcomes. Researchers observed that “an inconsistent and unpredictable number of children from at-risk populations presented with remarkably good mental and physical health outcomes despite the multiple disadvantages of structural, familial and individual stressors” (Ungar 2005, p. xvi). While there are different possible historical sources of resilience in the context of human development and behaviour across the realms of medicine, education and psychology, Masten (2007) points to the science of resilience arising in the human development field in the 1960s and 1970s and leading to developmental psychopathology. In fact, depending on one’s discipline, interests, and subjective stance towards the concept (e.g. whether one is a parent of a resilient teen or a mental health care provider for a teenager), perspectives can vary significantly. So while resilience is the subject of interest by different disciplines from distinct historical roots and conceptual perspectives, the potential policy strengths of resilience initiatives necessitate a firm understanding of its definition and concept.

Luthar and Brown (2007) refer to two core characteristics of the study of resilience. The first core attribute is that it is applied, with the goal of “understanding, and thereby ultimately well-being among those at risk” (p. 931). The second core attribute is that the study of resilience draws from methods and knowledge from different disciplinary fields. Most definitions of resilience have the same underlying recognition of the presence of adaptation/coping in response to risk/adversity/challenges (Barankin and Khanlou 2007; Joubart and Raeburn 1998; Masten 2001; Masten 2007; Masten, Herbers, Cutuli, and Lafavor 2008; Waller 2001). In order words, resilience develops in response to challenges, not in their absence, and the person (or system) can become stronger than before.

### Perspectives and Approaches to Resilience

Differing definitions and perspectives on resilience reflect the historical development of the concept, including the expansion of interests across various disciplines. Masten (2007) has suggested the field has experienced four waves of research with each wave leading to an evolution in breadth and complexity in our understanding and the implications for action. The first wave of research was descriptive and exploratory, seeking to capture measures of resilience in different forms and situations, and identifying characteristics of the child, family, relationships, wider environment, and the “short list of correlates” or factors thought to be protective from risk and promoting in the development of resilience. The second wave reflected an increasing sophistication in method and concept, emphasizing processes and longitudinality rather than traits or events in a single point in time. Increasingly, there was a widening and deepening of perspective. Over time, the need for action pressed forward the third wave of resilience research: a focus on the development and evaluation of programs that sought to promote or boost protective processes through pro-active intervention (the research of which forms the core of this review paper). Using randomized control studies or quasi-scientific methods, the wave of resilience research gaining momentum in the 1990s has helped forge the current array of evidence-based interventions.

Masten argues that there is an emergent fourth wave, one that reflects growing interest and knowledge of neural and psychobiological systems that might influence adaptative and resilient behaviour, areas such as brain plasticity. But Masten among others cautions against a singular over-emphasis on the “biology” of resilience. In doing so, she points to the enhanced sophistication in statistical method, computing and available data bases, and the emergence of multi-level and dynamic analysis. Together, these are expanding research into ways in which resilience might be shaped by diverse interactions across multiple levels of analysis such as gene-environment interaction, social interactions, social networks and person-media interactions (Masten 2007). From a multi-level analysis it is suggested an even more robust understanding of the interdependence of resilience with ecosystems, computer and communication systems, emergency systems, health care systems and political systems becomes increasingly possible. And from this, Masten suggests, an even stronger understanding of how dynamic human and policy systems might be shaped through cross-disciplinary findings.

With the evolutions in approach and knowledge brought forward by each wave of resilience research, three perspectives are evident in the literature:

Focus on individual factors: Originating from psychiatry and developmental psychology, the focus is on within-person factors and the notion of risk is important (Waller 2001: see ibid for a critique of individual, at-risk focus of earlier research on resilience and discussion of ecosystemic approach).
Constructionist approach: Postmodern interpretation, resilience is understood to be a result of negotiations between individuals and their environments, who self-define as healthy among conditions collectively viewed as adverse (Ungar 2004a, b).
Ecological, Ecosystemic approach: Informed by systems theory, relationships between risk and protective factors are considered, with emphasis on interdependency between individuals and social systems (Barankin and Khanlou 2007; Ungar 2004a; Waller 2001).

The eco-system approach is a useful perspective to consider resilience and resilience factors at the level of the individual, family and social environmental levels. The approach de-emphasizes an exclusive focus on the individual and their personal agency; instead, incorporating a more complex understanding of how individual factors and social factors interact to shape personal agency and structural opportunity (Evans 2007). From this perspective, public health interventions promoting resilience may be positioned to unite upstream (dealing with the broader determinants of resilience), midstream (for example focussing on parenting), and downstream (for example focussing on self-esteem) initiatives across sectors (including health, housing, education, employment training). Alperstein and Raman (2003) observe that with most of the research focusing on physical health, less research has considered upstream determinants of mental health concerns, “and even less on emotional well-being and enhancing ‘coping’ or promoting resilience,” (p. 269).

### Resilience: A Process and Multidimensional Concept

As our understanding of resilience grows, other viewpoints will emerge also adding to our knowledge-base in this area. For example, we argue for a *hybrid approach* that recognizes the value of combining multiple theoretical perspectives, epistemologies (positivistic and constructivist/interpretive/critical) in studying resilience, because in practice we need multidisciplinary and intersectoral approaches to promote resilience.

We believe it is helpful to consider resilience as a) a process (rather than a single event), b) a continuum (rather than a binary outcome), and c) likely a global concept with specific dimensions.

- *Process*: Resilience develops over time and depending on the interactions between systems involved, the time period can vary across individuals and settings.
- *Continuum*: Resilience has a continuum and the same person can be on different parts of the continuum of resilience depending on the support systems available and challenges faced over time.
- *Global*/*specific*: Resilience in the face of challenges can be experienced as a global process. However, it is likely that, similar to the concept of global and specific self-esteem (see Rosenberg, Schooler, Schoenbach, and Rosenberg 1995; Khanlou 2004), there are specific domains of resilience (for example, academic resilience, social resilience, etc.).^1^

### Factors associated with resilience: Individual, family and social environmental

Applying an ecosystemic approach, resilience promoting factors (also referred to in the literature as preventive factors) and resilience challenging factors (also referred to in the literature as risk factors) can be considered at the micro (individual), meso (family), and macro (environmental) levels. As per an ecosystemic approach, there are overlaps between the different levels, they interact with each other, and are not static in nature. However, for description purposes, it is helpful to present each separately, recognizing the dynamic nature of the relationships within and across the factors.

Resilience promoting and challenging factors have been categorized in different and overlapping ways in the literature. For example, Waller (2001) lists protective factors across ecosystemic levels under individual factors, family factors, community factors, and culture/ethnic identity. Here we follow the categorization and descriptions of Barankin and Khanlou (2007):

1. Individual factors
  As children grow into adolescents, and adolescents grow into young adults, they undergo developmental transitions. Individual factors entail temperament, learning strengths, self-concept, emotions, ways of thinking, adaptive skills, and social skills. A combination of each young person’s individual traits and their learning experiences gained through interactions with, and opportunities provided by, their family, school, and community help shape their resilience and the success with which developmental transitions are negotiated.^2^
2. Family factors
  The strengths that a family has and the challenges it faces change over time. These in turn interact with the individual level factors (discussed above) and are influenced by environmental factors (discussed below), impacting the resilience of each member of the family and the family as a whole. Family factors entail attachment, communication, parent relations, parenting style, and support outside the family.^3^
3. Social environmental factors
  Environmental factors include physical environments and social environments. Drawing from the growing body of research on the social determinants of health, the focus here is on social environments. Social environmental factors influence individual and family resilience factors. Embedded in them are the notions of fairness of opportunity, social justice, and mutual respect for all through practice, policies and laws (Barankin and Khanlou 2007). Social environmental factors entail social conditions, inclusion, access, and involvement.

### The Relationship between Resilience, Social Outcomes and Mental Health

Much of the academic and policy attention on the concept of resilience is driven by the association with a variety of social outcomes. The underlying empirical rationale for such attention is valid; there is a body of literature linking resilience and mental health status with social outcomes across the life course. Resilience (or the ‘lack’ thereof) has been associated with outcomes such as unsafe sex, poor educational performance and completion, bullying, crime, employment, job productivity and the likelihood of poverty are supported in research (Keyes 2004; Pressman and Cohen 2005; Scott et al. 2001; Sylva et al. 2007; Windle 2000). Equally so, is the recognition that many of these outcomes are “clustered” and often follow a social gradient patterned by socio-economic inequity.

Despite the evidence suggesting linkages between resilience and social outcomes such associations should be considered from the perspective of complexity rather than linearity and causal assumptions. Indeed, some resilience researchers force us to consider the uncomfortable, yet important, question of “what is a positive outcome”, particularly when weighed against other possible outcomes. As Ungar (2004a) and others remind us, it is quite possible to observe social behaviours that are typically constructed as “negative” or “poor”, yet are arguably quite adaptive to their social context. Moreover, measured gains in one domain of life might be contradicted by challenges in other domains. For example, an individual can achieve external positive social outcomes such as high levels of academic achievement, yet suffer from the effects of internal challenges such as anxiety and depression (Vanderbilt-Adriance and Shaw 2008).

Nor is the relationship between positive mental health and resilience clear, linear and uni-directional. Certainly enhancing resilience and promoting mental health can contribute significantly to healthier individuals and better social well-being, but it is not the whole story. How one defines mental health is varied and diverse. But within the variations there exist overlaps in relation to the perspective on life functioning, coping and control, and social connections. There is growing recognition that mental health is not the absence of mental illness. That a person with mental illness can experience positive mental health just as a person without a mental illness can have poor mental health (Friedli 2009). Mental health is linked to the capacity to flourish as opposed to languish. It is connected to relationships with families, with neighbours, with schools, and with work places (Lahtinen, Lehtinen, Riikonen and Ahonen 1999). More than just a state of mind, it is a positive state of being. Critical to such status are the assets and resources that one has access to cope with challenges, to take advantage of opportunities, and to negotiate a social position of respect, satisfaction and material well-being. From this perspective then, positive mental health is not exclusively internal and individual; it is also external and socially influenced.

What then is the connection between resilience and positive mental health? It would be too simple to suggest resilience is a ‘determinant’ of positive mental health or vice versa. Resilience and mental health are interlinked, overlapping, and bi-directional such that a young person with a mental health problem can be resilient or a resilient child or youth can develop a mental health problem (Barankin and Khanlou 2007). Nor is positive mental health the equivalent of a positive social outcome. A number of studies have reported findings of positive mental health from young persons who might be engaging in health risk activities or violent behaviour (Ungar 2004a). Instead, the complex process of resilience and the fostering of positive mental health are interactively conditioned by social context and economic circumstances shaping the environment in which individuals negotiate and sustain their definition of “healthy” (Ungar 2004a). Overall, the relationship between resilience, mental health and social outcomes are complex, non-linear and bi-directional.

### Evidence for Effective Policy and Interventions

Asking the simple question, ‘what works’, evokes at times more questions than it does answers. What works for whom? What are the desired effects and outcomes of interest? At what point in time do we conclude there has been a positive effect? To use the term resilience necessarily calls attention and caution in how resilience is perceived and constructed. Differing rates of prevalence conditioned by risk, observed stability and instability over time, and the lack of continuity of resilience across domains provide empirical support to the conceptual construction of resilience as a dynamic process over time, not a stable, static measurement (Vanderbilt-Adriance and Shaw 2008). Simply finding agreement on what to measure and when to measure it is a complex undertaking; nor should we expect to find evaluation evidence that is conclusively singular across the dimensions of context, time and domain. Just as there is no real effect as ‘ordinary magic’, it is necessary to avoid expectations of intervention as a ‘silver bullet’ or panacea.

Despite the prevalence of cautions, conditions and caveats cited above, there is growing strength in evidence that resilience-focused interventions can work (Adi et al. 2007; Green et al. 2005; Wells et al. 2003). Moreover, there are signs of widening consistency in what we know about resilience and what we know about the design of interventions, as well as growing convergence in the approaches and tools that make up the current mixed bag of resilience interventions. From this more optimistic perspective, it is possible to conclude that there is a sufficiently strong base of research and evaluation to promote, support and sustain resilience interventions.

Critical to the focus of this review is whether resilience interventions have sufficient strength and effectiveness to enhance an individuals’ capacity for overcoming social and economic adversity. In other words, do interventions hold promise towards helping to reduce socio-economic gaps in mental health and social outcomes? Is this promise evident across a variety of social categories such as gender, race, ethnicity, culture, income and neighbourhood conditions? To address these questions, we look at the evidence from three perspectives: i) whether interventions can improve ‘resilience’ for vulnerable children and youth; ii) whether there is a differential effect among different populations; and, iii) whether there is evidence that resilience interventions ‘close the gap’ on health and social outcome measures.

i) Strengthening resilience among vulnerable children and youth
  There is strong evidence for a number of programs towards improving the mental health and social wellbeing of children and youth exposed to high risk social circumstances. Many, if not most, of the programs were implemented and evaluated in areas of extreme, poverty, crime, and racial/ethnic concentration. The strength of this evidence is in favour of social emotional learning and similar mental health promotion orientations; there is less evidence for mental illness prevention programs (e.g., anxiety, depression) in relation to disadvantaged youth (Adi et al. 2007; Barry et al. 2009).
  While many of the studies lack longitudinal measures beyond 2 to 3 years, the most notable exception to this comes from the Seattle Development Project that has longitudinally tracked participants until the age twenty-one. The Seattle Development Project was a multi-component initiative comprised of three groups: 1) intervention for all students from Grades 1 through 6; and 2) a late 2 year intervention for all students in Grades 5-6; and, 3) a non-intervention control group. The program goal and outcomes were to improve functioning in school and work, enhance emotional and mental health, and reduce crime and substance use, and the project has been able to demonstrate significant positive effects at age 21 (Hawkins et al. 2005). The outcomes had a consistent ‘dose effect’; those engaged in the intervention for the longer period of time showed the most significant and lasting improvements, followed by those receiving the 2 year intervention. The study authors robustly conclude “the present results indicate that universal intervention during the elementary grades to strengthen teaching practices in public schools, strengthen parenting practices in multiethnic urban families, and ensure that children had the emotional and social skills to participate in the social life of elementary school had positive effects on functioning and mental health in early adulthood” (Hawkins et al. 2005, p. 30).
ii) Differential effect on populations
  Essential to understanding whether and how interventions might contribute to enhancing resilience and improving mental and social wellbeing for vulnerable and disadvantaged populations is determining if interventions might have differential effects. For example, in Canada with the “face” of social exclusion in Canada increasingly comprised of diverse populations along the lines of race, ethnicity and culture and with the long standing marginalization of Aboriginals, deconstructing and understanding how interventions might be influenced by these differences is of critical importance (Picot et al. 2007; Statistics Canada 2010). As well, there exists the possibility that gender might influence outputs and outcomes, either positively or negatively (Barry et al. 2009: Adi et al. 2007; Wells et al. 2003).
  - *Socio*-*Economic Background* - Some researchers have expressed the need to understand whether protective factors and interventions might in fact favour those from more advantaged socio-economic backgrounds (Vanderbilt-Adriance and Shaw 2008). The implication being that policy and action could in fact increase the SES gap in resilience and outcomes. The question is not whether children and youth can benefit from interventions–this was addressed in the previous section–the question is whether the impacts vary between advantaged and disadvantaged populations. Most existing studies are inadequate in design and power to provide strong evidence either way in relation to differential SES outcomes (Adi et al. 2007; Green et al. 2005; Wells et al. 2003).
  - *Race*, *Ethnicity and Culture*–The vast majority of studies have been limited in design and power to address how the effect of resilience interventions might differ in impact by race, ethnicity and culture (Adi et al. 2007). To some degree, these limitations from mixed population studies are off-set by other studies implemented in areas with a high racial concentration (e.g., African-Americans). However, evaluations drawn from economically deprived communities comprised, for example, of African-American and Latino populations tend to make no allowance for studying differential effects by population group (Lopez et al. 2002). Where some indications of differential cultural responses have been detected is in relation to mental health specific interventions (e.g. Penn Resiliency Model) where at least one study indicated no positive effect for low income African-American in Grade 5 and 6 (Cardemil et al. 2002). In response, there have been efforts to re-tailor the Penn Resiliency Model to more fully consider and adapt the intervention including such considerations as distinctions between cultural heterogeneity, the intersections of race/ethnicity and SES, and levels of acculturation and enculturation (Lopez et al. 2002).
  - *Gender–*The majority of studies do not adequately account for possible differential effects based on gender (Adi et al. 2007; Barry and Jenkins 2007). Where some differential is occasionally observed, the findings are generally mixed and unclear, often more closely related to smaller effect size rather than adverse impacts. Regardless, there remains a need to fill the research gap by more carefully considering different gender effects.
  In summary, there is a strong consensus in the evaluation field that future studies of resilience interventions must place a greater emphasis on differential population effects across the population domains of race, ethnicity, culture and gender (Adi et al. 2007; Ungar 2008).
iii) Closing the resilience gap between higher and lower income populations
  From this list of findings, challenges and issues, there remains the critical question of whether resilience interventions have the potential to close the health equity gap between advantaged and disadvantaged populations of children and youth. While there is good evidence that resilience interventions can have a positive impact on the mental and social well being of children and youth exposed to high risk social environments–in itself a good rationale for promoting resilience among children and youth–this is not the same as saying these enhancements actually “close the gap”. It has long been recognized among population health researchers it is quite possible to improve the well being of the most disadvantaged groups while the “gap” increases as more advantaged social groups achieve even larger improvements (Graham 2002). At this point in time, it is not possible to conclude that resilience interventions close the gap. When socio-economic and/or socio-cultural differences and improvements are captured in the evaluation research, the comparison group is their social peers rather than more advantaged groupings. Even in the case of robust longitudinal studies such as the Seattle Development Project, the control group and point of reference are those disadvantaged populations that did not receive the intervention. Therefore, while it is possible to conclude that a variety of resilience initiatives have improved the social outcomes and mental health of disadvantaged individuals and vulnerable groups, in the absence of comparisons with advantaged individuals it is not possible to assess whether these gains in fact narrowed differences in social outcomes.

## Discussion

Critical among external influences on mental health and resilience are the social determinants of health, from income and employment to physical environment and social environment. Over the last three decades, the field of population health and social determinants research has generated conceptual and empirical work enhancing our recognition that physical health is profoundly affected by factors such as income, education, the physical and social environment, gender and culture (Evans et al. 1994; Marmot and Wilkinson 1999; Heymann et al. 2006). Like physical health and illness, mental health and mental disorders are determined by multiple and interacting social, psychological, biological and environmental factors (Mrazek and Haggerty 1994; Desjarlais et al. 1995; Marmot and Wilkinson 1999; Hosman and Jané-Llopis 1999; Patel and Kleinman 2003; Patton et al. 1998; Friedli 2009). Internalized emotional and psychosocial factors are not simply derived from individual personality traits and, instead, are connected with the larger social environment (Barry et al. 2009). Explanations in the absence of considering more specific factors such as social position, social context, culture and other eco-system variables are only half explanations.

Few now question the significant contribution of social policy and social equity to better health status, mental health status and gaps in physical/mental health status. For thinking about resilience research and findings on resilience interventions, the essential requirement is how to position this knowledge in an eco-system perspective as in physical and mental health research. In developing a model for understanding the socially determined drivers and policy channels of health, Diderichsen and colleagues (2001) draw attention to the critical role of social stratification and social position. Social position will influence differential exposures to factors such as low income, unsafe neighbourhoods, etc. The first level of policy interventions then is to reduce stratification/disparity or the impact of disparity by minimizing exposure to these social risks, for example, through income assistance and other forms of social protection.

Yet exposure is not the same as vulnerability. Some people are more resistant to social adversity. Two persons in a position of low income do not necessarily suffer the same degree of damage to health, mental health or negative social consequences. Perhaps one has greater access to social support or lives in a neighbourhood with strong social capital. So while differential vulnerability is strongly influenced by social position and inequity, there are factors specific to the individual and their context that warrant attention.

Aspects of ‘differential vulnerability’ are strongly suggestive of resilience. That is, influencing differences in vulnerability to poor health or social outcomes is linked to policy interventions that enhance the personal agency of individuals to negotiate with and navigate within their social environment in over-coming negative exposures and adversity. Resilience research suggests that to increase one’s capacity to deter risk, build relationships and utilize their assets helps to reduce differential vulnerability–that the building of resilience and protective factors associated with resilience is one of the pathways to better health and well-being. But critical to this understanding is that strengthening resilience and reducing vulnerability is secondary to reducing exposures to adversity such as poverty and exclusion. In other words, fostering resilience is only one set of policy approaches in the social determinants ‘toolbox’.

Earlier we noted that the construct of resilience is complex and cuts across multiple domains of life. No single factor or attribute helps explain why some children and youth are able to overcome adversity, develop and maintain positive mental health and translate this gain into social accomplishment. Instead, resilience interventions such as school-based or community programs represent a pillar or one policy instrument. There is no solid evidence that such interventions alone are sufficient for closing the equity gap in mental and physical health, and the process of enhancing resilience and reducing social vulnerability will be limited when differential conditions of exposure remain large and intact. Social position and social conditions continue to carry a significant influence on the opportunities and outcomes at the individual and population level. The structured effects on race, ethnicity, culture, and gender will continue to bind and influence personal agency and potentially constrain choice and opportunity.

However, current social determinants analysis cannot always explain variations in outcome by disadvantaged social circumstances alone. Such disadvantage circumstances explain a great deal, but there remains considerable heterogeneity in outcomes. Indeed, it was examples drawn from such heterogeneity that led to the building of the resilience concept. Resilience research provides some indication of what might account for some proportion of this heterogeneity–differentials in vulnerability.

Within the literature, Seccombe (2002) has coined the phrases of “beating the odds” and “changing the odds” with the former referring to the process of resilience at an individual level and the latter in reference to changes in social risks and opportunities. “Beating the odds” accurately captures the process described throughout this paper of the factors and interventions that allow many individuals to overcome overwhelming social disadvantage. However, resilience research and evaluation findings on interventions both point towards limitations on the power of intervention through school, class, or community based programs. Even when such protective factors appear firmly in place, if the level of risk in the social environment is high then a lower rate of resilience is evident. In other words, protective factors “may not equally benefit children across various levels of risk” and are diminished (Vanderbilt-Adriance and Shaw 2008, p. 36). Ultimately, the higher the odds of social adversity, the less likelihood of an individual overcoming such adversity or “beating the odds”. Conversely, reducing risk and exposure lifts the likelihood for positive personal agency. So while we can talk colloquially and enthusiastically about “beating the odds”, citing anecdotal stories of the “magic” of resilience–research and evaluation provides ample evidence for reinforcing policy and action that “changes the odds”.

The implication is that programs and interventions designed to enhance resilience are *complimentary* within public health and population health–the effect does not *substitute* the need for consideration of income policy, housing policy, community development and other social determinants. A public health approach to promoting resilience should be grounded in the social determinants of health. Such a perspective does not isolate and segment “beating the odds” (differential vulnerability) and “changing the odds” (differential exposure).

### Whole Community Approach to Resilience: Closing the Public Health Policy Gap

Currently, there is a “policy gap” that needs to be filled (or completed) through the development and implementation of evidence-based resilience approaches marked by policy coherence, local integrated action, and community engagement. Research clearly indicates a wide range of powerful social influences and relationships that foster and promote the development of resilience–influences and relationships that cut across individual, family, community, school and society in a longitudinal process. Yet existing evidence-based programs and the diffusion of resilience interventions do not fully reflect such an expansive view of strength development, social risks and assets. Despite evidence pointing to the powerful influence of community interventions such as home visiting or early child development programs on improving life chances for disadvantaged or marginalized children, these critical developmental sources of resilience are not well defined or shaped by resilience research and content knowledge (at least in terms of well evaluated and explicit program philosophies and approaches such as social emotional learning). Nor are there readily available measurement tools for younger children as there are emerging for older children and youth. Filling this gap creates the potential for increasing the impact of early child development.

For the most part resilience initiatives tend to revolve within a single domain, with the most visible and active role played by schools. This “bias”, of course, should be placed in the appropriate context. The school and educational process of learning and growth represents the environment young persons’ spend the greatest proportion of their time outside of the family, and in a liberal democratic society, forms a critical basis for fostering full participatory citizenship. But it is not the only environment. The emergence of ‘whole school’ approach reflects one perspective in which resilience moves beyond just the class-room environment, further pushing the parameters by pointing to the need for partnerships beyond the boundaries of the school walls (Health Evidence Network 2006). And yet, when stepping back and taking a wide view perspective, the whole school approach continues to be driven from within one social domain–education.

**Fig. 1:**
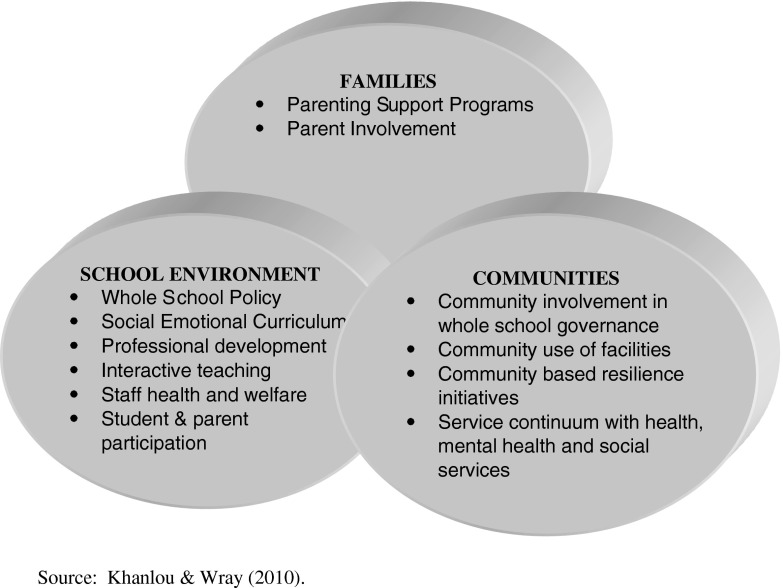
A ‘Whole community’ Approach of promoting resilience. *Source*: Khanlou and Wray (2010)

What is less evident is a coordinated multi-domain approach that fosters integration and coordination across child and youth developmental stages and between individual, family and social environment factors. In essence, there is an emerging need to expand the dimensions of resilience interventions with what we name as a ‘whole community’ approach to resilience. It builds on the concept of ‘whole school’ but recognizes that communities are more than schools and “ownership” of resilience should not be within schools only (which the “whole school” model suggests). A whole community approach is one in which the critical domains of resilience, family, school environment and community are integrated in the mission of fostering resilience through collaborative partnership and engagement Fig. 1.

### Promoting Resilience–A Process of Community Transformation

Filling the policy gap of promoting resilience is not about displacing other social policy and social initiatives such as the need for income policy. These resources remain critical determinants of successful healthy public policy and social support. Nor is building a Canadian approach of ‘whole community’ simply about new resources and funding–but using new local initiatives as a means of stimulating a wider transformation process.

While there is no quantitative research or even strong case studies in adopting a ‘whole community’ approach to resilience, there is certainly a large research base (and in many cases, substantial local experience) in community collaboration that can be adapted to foster a local transformation process. Helping to close the equity gap through the transformational and socially connecting process of fostering resilience and positive mental health is in the early stages. Some investments and activities are visible, a Canadian research base is emerging with international recognition, and interest across government sectors and within schools and communities is evolving. However, there is little Canadian research on effective interventions, with the bulk of the literature emerging from other jurisdictions, particularly the U.S. As well, additional work on considering differential effects among population groups and adapting existing evidence based interventions with tailored processes is a gap that should be closed.

## Conclusion

Overall, the evidence base for resilience is robust and the challenge is implementing and integrating established approaches and interventions in a Canadian and localized context. In moving toward localized approaches toward child and youth resilience promotion, research and practice can benefit from an intersectional understanding of child and youth development; as we have argued elsewhere: “intersectionality can position us to understand how agency (or resiliency and resourcefulness within the MHP [*Mental Health Promotion*] approach) is negotiated” across and within structures of society (Khanlou and Gonsalves 2011, p. 177). Intersections of each child’s or youth’s gender, lifestage, family socioeconomic resources and social networks within the context of their identity markers such as (but not limited to) ethnicity, racialized status, ability/disability, require individualized and localized approaches to resilience promotion. At the same time they require recognition of the broader determinants of health and wellbeing of populations. To this end, we believe the next wave of resilience promotion approaches and theory building would benefit from drawing from multiple disciplines including health, social sciences, and humanities.

## Footnotes

1 In the area of self-esteem, Rosenberg et al. (1995), consider global self-esteem as a general attitude toward the self and with more relevance to psychological wellbeing. They consider specific self-esteem as attitudes toward specific aspects of the self and with more relevance to behaviour.

2 In the original categorization by Barankin and Khanlou (2007) physical health was also listed under individual factors influencing children and youth.

3 In the original categorization by Barankin and Khanlou (2007) family structure, sibling’s relations, and parents’ health, was also listed under family factors influencing children and youth.
